# The complete chloroplast genome of *Cymbidium longibracteatum* (Orchidaceae) and phylogenetic analysis

**DOI:** 10.1080/23802359.2020.1780968

**Published:** 2020-07-23

**Authors:** Huijuan Ning, Lei Shao, Silan Dai

**Affiliations:** a College of Landscape Architecture, Beijing Forestry University, Beijing, China; b Zhejiang Provincial Key Laboratory of Germplasm Innovation and Utilization for Garden Plants, Key Laboratory of National Forestry and Grassland Administration on Germplasm Innovation and Utilization for Southern Garden Plants, School of Landscape Architecture, Zhejiang Agriculture & Forestry University, Hangzhou, China

**Keywords:** *Cymbidium longibracteatum*, chloroplast genome, Illumina sequencing, phylogeny

## Abstract

*Cymbidium longibracteatum* is a common cultivated species in the genus *Cymbidium* due to its elegant appearance, rich flower colors and strong fragrance, but its classification is quite controversial. In this study, the complete chloroplast genome of *C. longibracteatum* was obtained by Illumina sequencing. The chloroplast genome of *C. longibracteatum* is 150,070 bp in length with an overall GC content of 37.12%, which contains a large single-copy (LSC;84,949 bp) region, a small single-copy (SSC;13,745bp) region, and a pair of inverted repeats (IRs; 25,688 bp) regions. The genome contains 130 genes, namely 84 protein-coding genes, 38 tRNA genes and 8 rRNA genes. The maximum-likelihood phylogenetic tree has proved that *C. longibracteatum* should exist as an independent species in the genus *Cymbidium*, and it is most closely related to *C. tortisepalum*. This study provides valuable sequence resources for further study of *C. longibracteatum*.

*Cymbidium longibracteatum* (Orchidaceae) is mainly distributed in Sichuan, Guizhouand Yunnan Province of China, known as the authentic Sichuan orchid (Jie et al. 2013; Zhang et al. 2019). It exceeds most traditional and popular orchids owing to its elegant appearance, rich colors, and strong fragrance, which has high ornamental and economic value (Fengyan et al. 2009). Although *C. longibracteatum* is a common cultivated species in the genus *Cymbidium*, it is quite controversial in classification (Singchi and Zhongjian 2003). Yingsiang and Singchi (1980) classified *C. longibracteatum* as a variety of *C. goeringii*. Singchi and Zhongjian (2003), however, classified *C. longibracteatum* as a variety of *C. tortisepalum*. Due to the natural hybridization of the *Cymbidium* species in the nature, there are many intermediate types, making the boundaries of this species unclear (Ning et al. 2018). Today, the classification of *Cymbidium* species is mainly based on morphological indicators (Jiaping and Silan 1998). Meanwhile, there is also a lack of DNA data of the *Cymbidium* species, which is one of the most important tools in taxonomy (Ning et al. 2018). Therefore, it is urgent to provide valuable genetic information for this species. The complete chloroplast genome sequence can provide reliable data to identify species that are controversial in taxonomy, and it is shorter in length and more conservative in structure than the nuclear and mitochondrial genomes (Scarcelli et al. 2011). Here, we were the first to report the complete chloroplast genome of *C. longibracteatum*. This study will offer reliable molecular genetic data for the subsequent classification and identification of *Cymbidium*.

The mature leaves of *C. longibracteatum* were collected from Tang’jia mountain in Hongkou town, Bazhong city, Sichuan province, China (32°12′32.49″N; 107°58′26.89″E), and voucher specimen deposited at Orchid Resource Nursery of Zhejiang Agriculture and Forestry University (specimen code ZAFU20120218). Total genomic DNA was extracted by the modified CTAB method (Fu et al. 2017) and sequenced by NovaSeq platform (Illumina, USA). The clean reads were assembled by NOVOPlasty (Dierckxsens et al. 2017). The assembled sequence was annotated using CpGAVAS (Liu et al. 2012). The chloroplast genome map was generated using the online tool OGDRAW (Lohse et al. 2007). Finally, the complete chloroplast genome of *C. longibracteatum* was submitted to the GenBank (Accession Number: MT259022).

The chloroplast genome of *C. longibracteatum* is 150,070 bp in length with an overall GC content of 37.12%, which contains a large single-copy (LSC; 84,949 bp) region, a small single-copy (SSC; 13,745bp) region, and a pair of inverted repeats (IRs; 25,688 bp) regions. The genome encodes130 genes, namely 84 protein-coding genes, 38 tRNA genes, and 8rRNA genes.

To determine the phylogenetic position of *C. longibracteatum*, we selected 7 complete chloroplast genomes sequence of *Cymbidium* from NCBI GenBank for phylogenetic analysis. The sequences were aligned using MEGA X (Kumar et al. 2018) and the maximum-likelihood (ML) tree was constructed using RAxML v8.2.12 (Stamatakis, 2014) with 1000 bootstraps (Figure 1). The results indicated that *C. longibracteatum* should exist as an independent species in the genus *Cymbidium*, not as a variety of *C. goeringii* or *C. tortisepalum*, and it was sister to *C. tortisepalum*. The complete chloroplast genome of *C. longibracteatum* will contribute to further study of this species.

**Figure 1.**
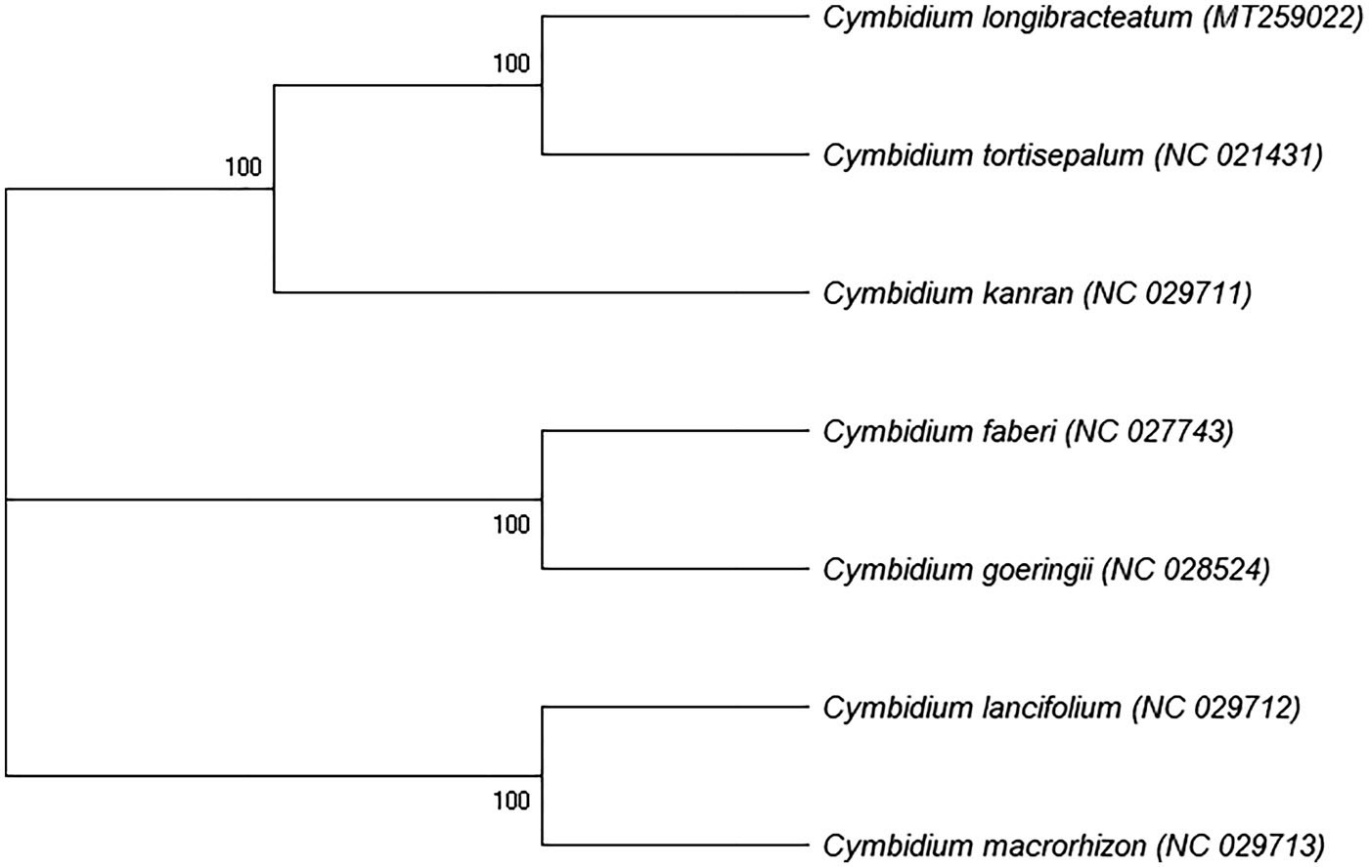
Maximum-likelihood phylogenetic tree based on 7 complete chloroplast genome sequences of *Cymbidium*. Numbers in the nodes indicate the bootstrap support values from 1000 replicates.

## Data Availability

The data that support the findings of this study are openly available in GenBank at https://www.ncbi.nlm.nih.gov, GenBank Accession Number: MT259022.
